# Solution to a Damped Duffing Equation Using He's Frequency Approach

**DOI:** 10.1155/2022/5009722

**Published:** 2022-07-11

**Authors:** Alvaro H. S. Salas, Gilder-Cieza Altamirano, Manuel Sánchez-Chero

**Affiliations:** ^1^Universidad Nacional de Colombia, Fizmako Research Group, Bogotá, Colombia; ^2^Universidad Nacional Autónoma de Chota, Cajamarca, Peru; ^3^Universidad Nacional de Frontera, Sullana, Peru

## Abstract

In this paper, we generalize He's frequency approach for solving the damped Duffing equation by introducing a time varying amplitude. We also solve this equation by means of the homotopy method and the Lindstedt–Poincaré method. High accurate formulas for approximating the Jacobi elliptic function cn are formally derived using Chebyshev and Pade approximation techniques.

## 1. Introduction

The deep understanding of the mechanism of nonlinear oscillations has an effective role in interpreting the ambiguities of many natural, physical, and engineering phenomena in various fields of science. Accordingly, many researchers have been able to give correct scientific explanations about their scientific experiences based on a deep understanding of the characteristics of these phenomena after the clarity of the ambiguity about the phenomenon under study. In the framework of nonlinear dynamics, there is no doubt that the scenario of dynamic mechanism of the pendulum motion is one of the objects that have deserved more attention in modeling all kind of phenomena related to oscillations, bifurcations, and chaos. The simple pendulum has been used as a physical model to solve problems such as nonlinear plasma oscillations, Duffing oscillators, Helmholtz oscillations, rigid plates that satisfy the Johanessen performance criteria, transverse vibrations nonlinear of a plate carrying a concentrated mass, a beam supported by a double periodic axial oscillating mount, cracks subjected to concentrated forces, surface waves in a plasma column, coupled modes of nonlinear bending vibrations of a circular ring, double spin spacecraft, motion of spacecraft over slowly rotating asteroids, nonlinear vibration of clasped beams, the nonlinear equation of wave, and nonlinear mathematical models of DNA.

It is known that the main objective of the numerical approaches is to find some numerical solutions to various realistic physical, engineering, and natural problems, especially when exact solutions are unavailable or extremely difficult to determine. There are many numerical approaches that were used for analyzing the family of the Duffing oscillator and Duffing–Helmholtz oscillator with constant coefficients. It is known that this family is integrable, i.e., its exact solution is available in the absence of the damping effect. On the other hand, if the damping effect and some other friction forces are taken into account, we get a nonintegral differential equation, i.e., its exact solution is not available.

The nonlinear oscillators have many applications in science and engineering. One of such oscillators is the Duffing equation. George Duffing, a German engineer, wrote a comprehensive book about this in 1918. Since then, there has been a tremendous amount of work done on this equation, including the development of solution methods (both analytical and numerical), and the use of these methods to investigate the dynamic behavior of physical systems that are described by the various forms of the Duffing equation.

Solution methods for nonlinear oscillators include the G/G method, modified mapping method and the extended mapping method, elliptic expansion method, modified (G/G)-expansion method, dynamical systems approach, the modified trigonometric function series method, generalized (G/G)-expansion method, tanh method, and the sn-ns method, among others [1–10].

In this paper, we consider the damped and unforced Duffing equation. We solve it using an extended version of He's frequency approach for the damped case. This oscillator was solved in [11] using a generalized elliptic functions. A simplification of the solution in [11] may be obtained by approximating the elliptic functions by means of trigonometric functions [12].

## 2. The Damped Duffing Oscillator

Let us consider the i.v.p.

(1) $$\begin{array}{c} \ddot{x}+2\varepsilon \dot{x}+\alpha x+\beta x^{3}=0,\;x\left(0\right)=x_{0}\text{ and }x^{\prime }\left(0\right)={\dot{x}}_{0}. \end{array}$$

The damped oscillator (1) is integrable only when *α*=8/9*ε*^2^. Equation (1) represents a damped Duffing oscillator. In the case when *ε*=0, we have an undamped Duffing oscillator which has an exact solution for any given arbitrary initial conditions. More precisely, the exact solution to the i.v.p.

(2) $$\begin{array}{c} \ddot{x}+\alpha x+\beta x^{3}=0,\;x\left(0\right)=x_{0}\text{ and }x^{\prime }\left(0\right)={\dot{x}}_{0}, \end{array}$$

is given by

(3) $$\begin{array}{c} x\left(t\right)=\frac{x_{0}cn\left(\sqrt{\omega }t|m\right)+b_{1}sn\left(\sqrt{\omega }t|m\right)dn\left(\sqrt{\omega }t|m\right)}{1+b_{2}sn^{2}\left(\sqrt{\omega }t|m\right)}, \end{array}$$

where

(4) $$\begin{array}{c} b_{1}=\frac{{\dot{x}}_{0}}{\sqrt{{\left(\alpha +\beta x_{0}^{2}\right)}^{2}+2\beta {\dot{x}}_{0}^{2}}},b_{2}=\frac{\alpha +\beta x_{0}^{2}}{2\sqrt{{\left(\alpha +\beta x_{0}^{2}\right)}^{2}+2\beta {\dot{x}}_{0}^{2}}}-\frac{1}{2},\\ \omega =\sqrt{{\left(\alpha +\beta x_{0}^{2}\right)}^{2}+2\beta {\dot{x}}_{0}^{2}},\text{ and }m=\frac{1}{2}-\frac{\alpha }{\sqrt{{\left(\alpha +\beta x_{0}^{2}\right)}^{2}+2\beta {\dot{x}}_{0}^{2}}},\\ {\left(\alpha +\beta x_{0}^{2}\right)}^{2}+2\beta {\dot{x}}_{0}^{2}\neq 0. \end{array}$$

The solution is periodic with period

(5) $$\begin{array}{c} T=\frac{4K\left(m\right)}{\sqrt{\omega }}, \end{array}$$

where

(6) $$\begin{array}{c} K\left(m\right)=\int_{0}^{\pi /2}\frac{\text{d}\theta }{\sqrt{1-m{\text{sin}}^{2}\theta }}. \end{array}$$

We may use the following approximation formulas for evaluating the elliptic integral *K*(*m*) (see Table 1).

See Table 2 for the approximation of 1/*K*(*m*).

For example,

(7) $$\begin{array}{c} x\left(t\right)=c_{0}\cos\left(\omega t+\;\cos^{-1}\left(\frac{x_{0}}{c_{0}}\right)\right), \end{array}$$

where

(8) $$\begin{array}{c} c_{0}=\pm \sqrt{x_{0}^{2}+\frac{{\dot{x}}_{0}^{2}}{\omega^{2}}},\omega =\frac{2\pi }{T},\\ T=\frac{4K\left(m\right)}{\sqrt{\alpha +\beta x_{0}^{2}}}\text{ and }m=\frac{\beta c_{0}^{2}}{2\left(\alpha +\beta c_{0}^{2}\right)}. \end{array}$$

Let us consider the i.v.p.

(9) $$\begin{array}{c} \ddot{x}+\alpha x+\beta x^{3}=0,x\left(0\right)=A\text{ and }x\prime \left(0\right)=0. \end{array}$$

A very good approximate analytical solution is given by

(10) $$\begin{array}{c} x\left(t\right)=A\frac{\sqrt{1+\lambda \text{cos}\left(\omega t\right)}}{\sqrt{1+\lambda {\text{cos}}^{2}\left(\omega t\right)}}, \end{array}$$

where

(11) $$\begin{array}{c} \omega =\frac{\pi \sqrt{\alpha +\beta A^{2}}}{2K\left(m\right)},\\ \;\lambda =\frac{\pi^{2}}{4K{\left(m\right)}^{2}}-1,\;\\ m=\frac{A^{2}\;\beta }{2\left(\alpha +A^{2}\beta \right)}. \end{array}$$

Now, observe that the function *x*(*t*)=cn(*t*, *m*) obeys the Duffing equation

(12) $$\begin{array}{c} \ddot{x}+\left(1-2m\right)x+2mx^{3}=0,x\left(0\right)=1\text{ and }x\prime \left(0\right)=0. \end{array}$$

Let *x*=*x*(*t*) be a continuous on [0,4*K*(*m*)] function. Define

(13) $$\begin{array}{c} E_{m}\left(x\right)=\max_{0\leq t\leq 4K\left(m\right)}\left|\text{cn}\left(t,m\right)-x\left(t\right)\right|. \end{array}$$

We have the following approximations for 0 ≤ *m* ≤ 0.5

(14) $$\begin{array}{c} \text{cn}\left(t,m\right)\approx x_{1}\left(t\right):=\cos\left(\frac{\pi }{2K\left(m\right)}t\right),\\ E_{m}\left(x_{1}\right)\approx \frac{13m}{149}+\frac{6m^{2}}{65}\leq 0.0674\text{ for }0\leq m\leq 0.5,\\ \text{cn}\left(t,m\right)\approx x_{2}\left(t\right):=\cos\left(\left(1-\frac{5m}{21}-\frac{3m^{2}}{23}\right)t\right),\\ E_{m}\left(x_{2}\right)\approx \frac{14m^{2}}{153}+\frac{17m}{186}\leq 0.06834\text{ for }0\leq m\leq 0.5,\\ cn\left(t,m\right)\approx x_{3}\left(t\right):=\frac{\sqrt{1-\left(4m/7\right)}\text{cos}\left(\left(-\left(3m^{2}/23\right)-\left(5m/21\right)+1\right)t\right)}{\sqrt{1-\left(4/7\right)m{\text{cos}}^{2}\left(\left(-\left(3m^{2}/23\right)-\left(5m/21\right)+1\right)t\right)}},\\ E_{m}\left(x_{3}\right)\approx \frac{m}{100}-\frac{m^{2}}{210}\leq 0.0089\text{ for }0\leq m\leq 0.5,\\ \text{cn}\left(t,m\right)\approx x_{4}\left(t\right):=\frac{\sqrt{1-\left(2143m/3564\right)}\text{cos}\left(\left(-\left(10m^{2}/77\right)-\left(11m/46\right)+1\right)t\right)}{\sqrt{1-\left(53/81\right)m{\text{cos}}^{2}\left(\left(-\left(10m^{2}/77\right)-\left(11m/46\right)+1\right)t\right)+\left(7/132\right)m{\text{cos}}^{4}\left(\left(-\left(10m^{2}/77\right)-\left(11m/46\right)+1\right)t\right)}},\\ E_{m}\left(x_{4}\right)\approx \frac{2m}{91}-\frac{4m^{2}}{89}\leq 0.0045\text{ for }0\leq m\leq 0.5,\\ \text{cn}\left(t,m\right)\approx x_{5}\left(t\right):=\left(1-\frac{m}{10}\right)\cos\left(\left(1-\frac{5m}{21}-\frac{3m^{2}}{23}\right)t\right)+\frac{m}{10}\text{cos}\left(3\left(1-\frac{5m}{21}-\frac{3m^{2}}{23}\right)t\right),\\ E\left(x_{5}\right)\approx \frac{5m}{69}-\frac{11m^{2}}{109}\leq 0.0153\text{ for }0\leq m\leq 0.5,\\ \text{cn}\left(t,m\right)\approx x_{6}\left(t\right):=\left(1-\frac{131m}{1500}\right)\text{cos}\left(\left(-\frac{3m^{2}}{23}-\frac{5m}{21}+1\right)t\right)+\frac{m}{12}\text{cos}\left(3\left(-\frac{3m^{2}}{23}-\frac{5m}{21}+1\right)t\right)+\frac{m}{250}\cos\left(5\left(-\frac{3m^{2}}{23}-\frac{5m}{21}+1\right)t\right),\\ E_{m}\left(x_{6}\right)\approx \frac{m}{23}-\frac{5m^{2}}{62}\leq 0.00781\text{ for }0\leq m\leq 0.5.\\ \; \end{array}$$

The approximations above may be used to give trigonometric solution to the i.v.p. (9) whose exact solution reads

(15) $$\begin{array}{c} x\left(t\right)=\text{Acn}\left(\sqrt{\alpha +\beta A^{2}t},\frac{\beta A^{2}}{2\left(\alpha +\beta A^{2}\right)}\right). \end{array}$$

For example,

(16) $$\begin{array}{c} x\left(t\right)=A\text{cos}\left(\left(1-\frac{10}{33}\left(\frac{\beta A^{2}}{2\left(\alpha +\beta A^{2}\right)}\right)\right)\sqrt{\alpha +\beta A^{2}t}\right). \end{array}$$

The frequency-amplitude formulation for this solution is given by

(17) $$\begin{array}{c} \omega^{2}=\frac{{\left(33\alpha +28A^{2}\beta \right)}^{2}}{1089\left(\alpha +A^{2}\beta \right)}. \end{array}$$

We obtain several frequency-amplitude formulations using formulas. The He's frequency-amplitude formulation for the Duffing equation $\ddot{x}+\alpha x+\beta x^{3}=0$ establishes that

(18) $$\begin{array}{c} \omega_{0}^{2}=w\prime {\left(t\right)}^{2}=\alpha +\frac{3}{4}\beta A^{2},\text{ where }w\left(t\right)=\sqrt{\alpha +\frac{3}{4}\beta A^{2}t}. \end{array}$$

## 3. He's Approach

Using He's frequency approach, an approximate analytical solution in the absence of damping (*ε*=0) may be obtained using

(19) $$\begin{array}{c} x_{\text{approx}}\left(t\right)=A\text{cos}\left(\sqrt{\omega }t+{\text{cos}}^{-1}\left(\frac{x_{0}}{A}\right)\right),\\ A=\pm \sqrt{\frac{-4\alpha +3\beta x_{0}^{2}\pm \sqrt{{\left(4\alpha +3\beta x_{0}^{2}\right)}^{2}+48\beta {\dot{x}}_{0}^{2}}}{6\beta }}\text{ and }\omega =\alpha +\frac{3\beta }{4}A^{2}. \end{array}$$

Let

(20) $$\begin{array}{c} w\left(t\right)=\sqrt{\omega }t. \end{array}$$

Observe that *w*(0)=0. He's idea for the undamped case is based on the following fact:

(21) $$\begin{array}{c} w\prime {\left(t\right)}^{2}=\omega_{0}^{2}=\frac{f\left(x\right)}{x}=\alpha +\frac{3\beta }{4}A^{2}\text{ for }x=\frac{\sqrt{3}}{2}A\text{ and }f\left(x\right)=\alpha x+\beta x^{3}. \end{array}$$

Following this idea, for the damped case, we will replace *A* with *A*exp(−*ρt*) so that

(22) $$\begin{array}{c} w\prime {\left(t\right)}^{2}=\alpha +\frac{3\beta }{4}A^{2}\text{exp}\left(-2\rho t\right)\text{ and }w\left(0\right)=0. \end{array}$$

The approximate analytical solution for the damped Duffing equation (1) will then be

(23) $$\begin{array}{c} x_{\text{approx}}\left(t\right)=A\text{exp}\left(-\rho t\right)\text{cos}\left(w\left(t\right)+{\text{cos}}^{-1}\left(\frac{x_{0}}{A}\right)\right). \end{array}$$

From (22) it follows that

(24) $$\begin{array}{c} w\left(t\right)=\int_{0}^{t}\sqrt{\alpha +\frac{3\beta }{4}A^{2}\text{exp}\left(-2\rho \tau \right)}\text{d}\tau =W\left(t\right)-W\left(0\right), \end{array}$$

where

(25) $$\begin{array}{c} W\left(t\right)=\frac{\sqrt{4\alpha +3A^{2}\beta e^{-2\rho t}}}{2A\rho }\left(\frac{2\sqrt{\alpha }Ae^{\rho t}}{\sqrt{3A^{2}\beta +4\alpha e^{2\rho t}}}\text{sin}h^{-1}\left(\frac{2\sqrt{\alpha }e^{\rho t}}{\sqrt{3}A\sqrt{\beta }}\right)-A\right). \end{array}$$

The number *ρ* is a free parameter that is chosen in order to get as small residual error as possible. The default value is *ρ*=*ε*.

## 4. Homotopy Perturbation Method

We seek a solution in the ansatz form:

(26) $$\begin{array}{c} x\left(t\right)=v_{0}\left(t\right)+pv_{1}\left(t\right)+p^{2}v_{2}\left(t\right)+\cdots . \end{array}$$

The homotopy is defined as follows:

(27) $$\begin{array}{c} H\left(x,p\right)=\ddot{x}+2\varepsilon \dot{x}+\alpha x+p\beta x^{3}. \end{array}$$

We have

(28) $$\begin{array}{c} H\left(x,p\right)=\alpha v_{0}\left(t\right)+2\varepsilon v_{0}^{\prime }\left(t\right)+v_{0}^{\prime \prime }\left(t\right)+\left(\alpha v_{1}\left(t\right)+\beta v_{0}{\left(t\right)}^{3}+2\varepsilon v_{1}^{\prime }\left(t\right)+v_{1}^{\prime \prime }\left(t\right)\right)p+\left(\alpha v_{2}\left(t\right)+3\beta v_{1}\left(t\right)v_{0}{\left(t\right)}^{2}+2\varepsilon v_{2}^{\prime }\left(t\right)+v_{2}^{\prime \prime }\left(t\right)\right)p^{2}+\cdots . \end{array}$$

We now equate to zero the coefficients of *p*^*j*^(*j*=0,1,2,3,…) and then we obtain an ode system. We solve these odes so that the functions *v*_*j*_(*t*)(*j*=1,2,3,…) do not contain secularity terms. The solutions are

(29) $$\begin{array}{c} v_{0}\left(t\right)=c_{0}e^{-\varepsilon \left(c_{1}+t\right)}\text{cos}\left(\sqrt{\alpha -\varepsilon^{2}}\left(c_{1}+t\right)\right),\\ v_{1}\left(t\right)=\frac{\beta c_{0}^{3}e^{-3\varepsilon }\left(c_{1}+t\right)}{16\alpha \sqrt{\alpha -\varepsilon^{2}}\left(3\varepsilon^{2}-4\alpha \right)}\left(\begin{array}{c} 3\varepsilon \left(\varepsilon^{2}-\alpha \right)\text{sin}\left(3\sqrt{\alpha -\varepsilon^{2}}\left(c_{1}+t\right)\right)+\\ \sqrt{\alpha -\varepsilon^{2}}\left(3\varepsilon^{2}-2\alpha \right)\cos\left(3\sqrt{\alpha -\varepsilon^{2}}\left(c_{1}+t\right)\right) \end{array}\right),\\ v_{2}\left(t\right)=\frac{3\beta^{2}c_{0}^{5}e^{-5\varepsilon \left(c_{1}+t\right)}}{512\alpha^{2}\left(3\varepsilon^{2}-4\alpha \right)\left(\alpha +3\varepsilon^{2}\right)\left(5\varepsilon^{2}-9\alpha \right)}\\ \left(\begin{array}{c} -4\left(5\varepsilon^{2}-9\alpha \right)\left(\alpha^{2}-9\alpha \varepsilon^{2}+9\varepsilon^{4}\right)\text{cos}\left(3\sqrt{\alpha -\varepsilon^{2}\left(c_{1}+t\right)}\right)\\ +2\left(\alpha +3\varepsilon^{2}\right)\left(3\alpha^{2}-17\alpha \varepsilon^{2}+15\varepsilon^{4}\right)\cos\left(5\sqrt{\alpha -\varepsilon^{2}\left(c_{1}+t\right)}\right)\\ +\varepsilon \sqrt{\alpha -\varepsilon^{2}}\left(\begin{array}{c} 18\left(2\varepsilon^{2}-\alpha \right)\left(5\varepsilon^{2}-9\alpha \right)\text{sin}\left(3\sqrt{\alpha -\varepsilon^{2}}\left(c_{1}+t\right)\right)-\\ \left(\alpha +3\varepsilon^{2}\right)\left(30\varepsilon^{2}-19\alpha \right)\text{sin}\left(5\sqrt{\alpha -\varepsilon^{2}}\left(c_{1}+t\right)\right) \end{array}\right) \end{array}\right) \end{array}$$

The numbers *c*_0_ and *c*_1_ are obtained from the initial conditions *x*(0)=*x*_0_ and $x\prime \left(0\right)={\dot{x}}_{0}$.

## 5. Improved Analytical Solution

### 5.1. First Approach

Assume the ansatz

(30) $$\begin{array}{c} x\left(t\right)=A\text{exp}\left(-\rho t\right)\text{cos}\left(\omega \left(t\right)+{\text{cos}}^{-1}\left(\frac{x_{0}}{A}\right)\right),f\left(0\right)=0. \end{array}$$

Then,

(31) $$\begin{array}{c} \ddot{x}+2\varepsilon \dot{x}+\alpha x+\beta x^{3}=\frac{1}{4}A^{3}\beta \;\cos\left(3\theta \right)e^{-3\rho t}+\frac{1}{4}A\;\cos\left(\theta \right)e^{-3\rho t}\left(3A^{2}\beta -4e^{2\rho t}\omega \prime {\left(t\right)}^{2}+4e^{2\rho t}\left(\alpha -2\varepsilon \rho +\rho^{2}\right)\right)-A\;\sin\left(\theta \right)e^{\rho \left(-t\right)}\left(\omega \prime \prime \left(t\right)+2\left(\varepsilon -\rho \right)\omega \prime \left(t\right)\right). \end{array}$$

We will choose the function *ω*=*ω*(*t*) so that

(32) $$\begin{array}{c} 3A^{2}\beta -4e^{2\rho t}\omega \prime {\left(t\right)}^{2}+4e^{2\rho t}\left(\alpha -2\varepsilon \rho +\rho^{2}\right)=0\text{ and }\omega \left(0\right)=0, \end{array}$$

then,

(33) $$\begin{array}{c} \omega \left(t\right)=\int_{0}^{t}\sqrt{\alpha -2\varepsilon \rho +\rho^{2}+\frac{3}{4}A^{2}\beta e^{-2\rho \tau }}\text{d}\tau =\Omega \left(t\right)-\Omega \left(0\right), \end{array}$$

where

(34) $$\begin{array}{c} \Omega \left(t\right)=\frac{1}{\rho }\left(\sqrt{\alpha -2\varepsilon \rho +\rho^{2}}\text{tan}h^{-1}\left(\sqrt{\frac{\alpha -2\varepsilon \rho +\rho^{2}+\left(3/4\right)A^{2}e^{-2t\rho }\beta }{\alpha -2\varepsilon \rho +\rho^{2}}}\right)-\sqrt{\alpha -2\varepsilon \rho +\rho^{2}+\frac{3}{4}A^{2}e^{-2t\rho }\beta }\right). \end{array}$$

Observe that

(35) $$\begin{array}{c} \omega \prime {\left(t\right)}^{2}=\alpha -2\varepsilon \rho +\rho^{2}+\frac{3}{4}A^{2}\beta e^{-2\rho t}=\frac{f\left(x\right)}{x}\text{ for }x=\frac{\sqrt{3}}{2}A\text{exp}\left(-\rho t\right),f\left(x\right)=\left(\alpha -2\varepsilon \rho +\rho^{2}\right)x+\beta x^{3}. \end{array}$$

In other words, when *ε*=*ρ*=0, this corresponds to the He's frequency formulation for the Duffing equation:

(36) $$\begin{array}{c} \ddot{x}+\left(\alpha -2\varepsilon \rho +\rho^{2}\right)x+\beta x^{3}=0. \end{array}$$

The number *ρ* is chosen in order to minimize the residual error. A default value for *ρ* is obtained by eliminating *A* from the system:

(37) $$\begin{array}{c} \omega \prime \prime \left(0\right)+2\left(\varepsilon -\rho \right)\omega \prime \left(0\right)=0\text{ and }x\prime \left(0\right)={\dot{x}}_{0}. \end{array}$$

This last condition gives the sextic:

(38) $$\begin{array}{c} 12\beta \varepsilon^{2}{\dot{x}}_{0}^{2}-2\varepsilon \left(4\alpha^{2}+3\alpha \beta x_{0}^{2}-12\beta \varepsilon x_{0}{\dot{x}}_{0}+18\beta {\dot{x}}_{0}^{2}\right)\rho +\left(8\alpha^{2}+32\alpha \varepsilon^{2}+9\alpha \beta x_{0}^{2}+24\beta \varepsilon^{2}x_{0}^{2}-72\beta \varepsilon x_{0}{\dot{x}}_{0}+27\beta {\dot{x}}_{0}^{2}\right)\rho^{2}-2\left(24\alpha \varepsilon +16\varepsilon^{3}+30\beta \varepsilon x_{0}^{2}-27\beta x_{0}{\dot{x}}_{0}\right)\rho^{3}+4\left(4\alpha +16\varepsilon^{2}+9\beta x_{0}^{2}\right)\rho^{4}-40\varepsilon \rho^{5}+8\rho^{6}=0. \end{array}$$

A root to this sextic near *ρ*=*ε* may be evaluated using the following approximation:

(39) $$\begin{array}{c} \rho \approx \varepsilon -\frac{\begin{array}{c} 6\beta \varepsilon \left(\alpha x_{0}^{2}+2\varepsilon {\dot{x}}_{0}x_{0}+{\dot{x}}_{0}^{2}\right)\left(4{\left(\alpha -\varepsilon^{2}\right)}^{2}+6\beta x_{0}^{2}\left(\alpha +\varepsilon^{2}\right)+21\beta \varepsilon x_{0}{\dot{x}}_{0}+9\beta {\dot{x}}_{0}^{2}\right) \end{array}}{64{\left(\alpha -\varepsilon^{2}\right)}^{4}+3\beta \left(3\beta x_{0}^{4}\left(13\alpha^{2}+12\alpha \varepsilon^{2}+16\varepsilon^{4}\right)+4\varepsilon {\dot{x}}_{0}x_{0}\left(52{\left(\alpha -\varepsilon^{2}\right)}^{2}+x_{0}^{2}\left(57\alpha \beta +54\beta \varepsilon^{2}\right)\right)+4{\dot{x}}_{0}^{2}\left(22{\left(\alpha -\varepsilon^{2}\right)}^{2}+3\beta x_{0}^{2}\left(9\alpha +41\varepsilon^{2}\right)\right)+8x_{0}^{2}{\left(\alpha -\varepsilon^{2}\right)}^{2}\left(7\alpha +8\varepsilon^{2}\right)+360\beta \varepsilon {\dot{x}}_{0}^{3}x_{0}+81\beta {\dot{x}}_{0}^{4}\right)}. \end{array}$$

The value of *A* is given by

(40) $$\begin{array}{c} A=\pm \sqrt{3\beta x_{0}^{2}+8\varepsilon \rho -4\alpha -4\rho^{2}\pm \frac{\sqrt{{\left(4\left(\alpha +\rho \left(\rho -2\varepsilon \right)\right)-3\beta x_{0}^{2}\right)}^{2}+48\beta \left(x_{0}^{2}\left(\alpha +2\rho \left(\rho -\varepsilon \right)\right)+2\rho {\dot{x}}_{0}x_{0}+{\dot{x}}_{0}^{2}\right)}}{6\beta }}. \end{array}$$

### 5.2. Second Approach

The exact solution to the undamped Duffing equation $\ddot{x}+\alpha x+\beta x^{3}=0$ is given by

(41) $$\begin{array}{c} x\left(t\right)=c_{0}\text{cn}\left(w\left(t\right)+c_{1},m\right), \end{array}$$

where

(42) $$\begin{array}{c} w\left(t\right)=\sqrt{\alpha +\beta c_{0}^{2}t}\text{ and }m=\frac{\beta c_{0}^{2}}{2\left(\alpha +\beta c_{0}^{2}\right)}. \end{array}$$

Then,

(43) $$\begin{array}{c} w\prime {\left(t\right)}^{2}=\alpha +\beta c_{0}^{2}\text{ and }m=\frac{\beta c_{0}^{2}}{2w\prime {\left(t\right)}^{2}}. \end{array}$$

Let us replace  *α* with *α* − 2*ρε*+*ρ*^2^ and *c*_0_ with *c*_0_exp(−*ρt*) so that

(44) $$\begin{array}{c} w\prime {\left(t\right)}^{2}=\alpha -2\rho \varepsilon +\rho^{2}+\beta c_{0}^{2}\text{exp}\left(-2\rho t\right)\text{ and }m=m\left(t\right)=\frac{\beta c_{0}^{2}\text{exp}\left(-2\rho t\right)}{2w\prime {\left(t\right)}^{2}}=\frac{\left(1/2\right)}{1+\left(\alpha -2\rho \varepsilon +\rho^{2}/\beta c_{0}^{2}\right)\text{exp}\left(2\rho t\right)}. \end{array}$$

For the damped Duffing equation we will define the solution in the form:

(45) $$\begin{array}{c} x\left(t\right)=c_{0}\text{exp}\left(-\rho t\right)\text{cn}\left(\lambda \int_{0}^{t}\sqrt{\alpha -2\rho \varepsilon +\rho^{2}+\beta c_{0}^{2}\text{exp}\left(-2\rho t\right)\;}d\tau +c_{1},\frac{\left(1/2\right)}{1+\left(\alpha -2\rho \varepsilon +\rho^{2}/\beta c_{0}^{2}\right)\text{exp}\left(2\rho t\right)}\right). \end{array}$$

The constants *c*_0_ and *c*_1_ are determined from the initial conditions. On the other hand,

(46) $$\begin{array}{c} \int_{0}^{t}\sqrt{\alpha -2\rho \varepsilon +\rho^{2}+\beta c_{0}^{2}\exp\left(-2\rho t\right)}\text{d}\tau =W\left(t\right)-W\left(0\right), \end{array}$$

where

(47) $$\begin{array}{c} W\left(t\right)=\frac{1}{\rho }\left(\sqrt{\alpha -2\varepsilon \rho +\rho^{2}}\text{tan}h^{-1}\left(\sqrt{\frac{\beta c_{0}^{2}e^{-2\rho t}}{\alpha -2\varepsilon \rho +\rho^{2}}+1}\right)-\sqrt{\alpha +\beta c_{0}^{2}e^{-2\rho t}-2\varepsilon \rho +\rho^{2}}\right). \end{array}$$

The numbers *ρ* and *λ* are free parameters that we choose in order to get as small residual error as possible. The default *ρ* value is *ρ*=(2/3*ε*) and the default value for *λ* is *λ*=1. Observe that this ansatz will give the exact solution for the integrable case, i.e., when *ρ*=(2/3*ε*) and *α*=(8/9*ε*^2^).

## 6. Lindstedt–Poincaré Method

We seek a solution in the ansatz form:

(48) $$\begin{array}{c} x\left(t\right)=u_{0}\left(t\right)+\beta u_{1}\left(t\right)+\beta^{2}u_{2}\left(t\right)+\cdots , \end{array}$$

then.

(49) $$\begin{array}{c} \ddot{x}++2\varepsilon \dot{x}+\alpha x+\beta x^{3}=\alpha u_{0}\left(t\right)+2\varepsilon u_{0}^{\prime }\left(t\right)+u_{0}^{\prime \prime }\left(t\right)+\left(\alpha u_{1}\left(t\right)+2\varepsilon u_{1}^{\prime }\left(t\right)+u_{1}^{\prime \prime }\left(t\right)+u_{0}{\left(t\right)}^{3}\right)\beta +\left(\alpha u_{2}\left(t\right)+2\varepsilon u_{2}^{\prime }\left(t\right)+u_{2}^{\prime \prime }\left(t\right)+3u_{1}\left(t\right)u_{0}{\left(t\right)}^{2}\right)\beta^{2}+\cdots . \end{array}$$

We now equate to zero the coefficients of *β*^*j*^(*j*=0,1,2,3,…) and then we obtain an ode system. We solve these odes so that the functions *u*_*j*_(*t*)(*j*=1,2,3,…) do not contain secularity terms. The solutions are

(50) $$\begin{array}{c} u_{0}\left(t\right)=c_{0}e^{-\varepsilon \left(c_{1}+t\right)}\text{cos}\left(\sqrt{\alpha -\varepsilon^{2}}\left(c_{1}+t\right)\right),\\ u_{1}\left(t\right)=\frac{c_{0}^{3}e^{-3\varepsilon \left(c_{1}+t\right)}}{16\alpha \left(3\varepsilon^{2}-4\alpha \right)}\left(\left(3\varepsilon^{2}-2\alpha \right)\text{cos}\left(3\sqrt{\alpha -\varepsilon^{2}}\left(c_{1}+t\right)\right)-3\varepsilon \sqrt{\alpha -\varepsilon^{2}}\sin\left(3\sqrt{\alpha -\varepsilon^{2}}\left(c_{1}+t\right)\right)\right),\\ u_{2}\left(t\right)=\frac{3c_{0}^{5}e^{-5\varepsilon \left(c_{1}+t\right)}\left(-4\left(5\varepsilon^{2}-9\alpha \right)\left(\alpha^{2}-9\alpha \varepsilon^{2}+9\varepsilon^{4}\right)\cos\left(3\sqrt{\alpha -\varepsilon^{2}}\left(c_{1}+t\right)\right)+2\left(\alpha +3\varepsilon^{2}\right)\left(3\alpha^{2}-17\alpha \varepsilon^{2}+15\varepsilon^{4}\right)\cos\left(5\sqrt{\alpha -\varepsilon^{2}}\left(c_{1}+t\right)\right)+\varepsilon \sqrt{\alpha -\varepsilon^{2}}\left(18\left(2\varepsilon^{2}-\alpha \right)\left(5\varepsilon^{2}-9\alpha \right)\sin\left(3\sqrt{\alpha -\varepsilon^{2}}\left(c_{1}+t\right)\right)-\left(\alpha +3\varepsilon^{2}\right)\left(30\varepsilon^{2}-19\alpha \right)\sin\left(5\sqrt{\alpha -\varepsilon^{2}}\left(c_{1}+t\right)\right)\right)\right)}{512\alpha^{2}\left(3\varepsilon^{2}-4\alpha \right)\left(\alpha +3\varepsilon^{2}\right)\left(5\varepsilon^{2}-9\alpha \right)}. \end{array}$$

The constants *c*_0_ and *c*_1_ are obtained from the initial conditions.

## 7. Numerical Solution

We make use of the following backward finite differences formulas for the first and second derivatives:

(51) $$\begin{array}{c} \dot{x}\left(t_{i}\right)\approx \frac{3x_{i-4}-16x_{i-3}+36x_{i-2}-48x_{i-1}+25x_{i}}{12\Delta t}\text{ for }i\geq 4,\\ \ddot{x}\left(t_{i}\right)\approx \frac{11x_{i-4}-56x_{i-3}+114x_{i-2}-104x_{i-1}+35x_{i}}{12\Delta t^{2}}\text{ for }i\geq 4. \end{array}$$

The discretized ode reads

(52) $$\begin{array}{c} \frac{11x_{i-4}-56x_{i-3}+114x_{i-2}-104x_{i-1}+35x_{i}}{12\Delta t^{2}}+2\varepsilon \frac{3x_{i-4}-16x_{i-3}+36x_{i-2}-48x_{i-1}+25x_{i}}{12\Delta t}+\alpha x_{i}+\beta x_{i}^{3}=0\;i=4,5,6,\ldots ,n,\;n=\lfloor \frac{T}{\Delta t}\rfloor ,\;t_{i}=i\cdot \Delta t. \end{array}$$

The values *x*_1_=*x*(*t*_1_), *x*_2_=*x*(*t*_2_), and *x*_3_=*x*(*t*_3_) are obtained from some reasonable analytical or numerical solution. The value *x*_4_ is found from the cubic:

(53) $$\begin{array}{c} \beta x_{4}^{3}+\left(\alpha +\frac{5\left(10\varepsilon \Delta t+7\right)}{12\Delta t^{2}}\right)x_{4}+\frac{x_{0}\left(6\varepsilon \Delta t+11\right)-8x_{1}\left(4\varepsilon \Delta t+7\right)+6x_{2}\left(12\varepsilon \Delta t+19\right)-8x_{3}\left(12\varepsilon \Delta t+13\right)}{12\Delta t^{2}}=0. \end{array}$$

We choose the closest to *x*_3_ real root to the cubic in equation (53). Suppose we already found the values *x*_*k*_ for *k*=4,5,…, *i* − 1. Then, the value of *x*_*i*_ is obtained by choosing the closest to *x*_*i*−1_ real root to the cubic in equation (52). Thus, we solve the ode recursively using Tartaglia's formula for the cubic. For small Δ*t*, we may use the following practical formula:

(54) $$\begin{array}{c} x_{i}=x_{i-1}-\frac{\left(p+3x_{i-1}^{2}\right)\left(x_{i-1}^{3}+px_{i-1}+q\right)}{p^{2}-3qx_{i-1}3px_{i-1}^{2}+6x_{i-1}^{4}}, \end{array}$$

where

(55) $$\begin{array}{c} p=\frac{\left(6\varepsilon \Delta t+11\right)x_{i-4}-8\left(4\varepsilon \Delta t+7\right)x_{i-3}+6\left(12\varepsilon \Delta t+19\right)x_{i-2}-8\left(12\varepsilon \Delta t+13\right)x_{i-1}}{12\beta \Delta t^{2}},\\ q=\frac{12\alpha \Delta t^{2}+50\varepsilon \Delta t+35}{12\beta \Delta t^{2}}. \end{array}$$

**Remark 1 .**

We also may use the following formulas based on the Runge–Kutta method.

(56) $$\begin{array}{c} x_{i}=x_{i-1}+\frac{\Delta {\text{t}}^{2}}{6}\left(f\left(\frac{\Delta \text{t}}{2}+x_{i-1},\frac{1}{2}\Delta \text{t}x_{i-1}+x_{i-1}\frac{1}{2}\Delta \text{t}f\left(x_{i-1},x_{i-1},x_{i-1}\right)+x_{i-1}\right)+f\left(\frac{\Delta \text{t}}{2}+x_{i-1},\frac{1}{4}\Delta \text{t}\left(\Delta \text{t}f\left(x_{i-1},x_{i-1},x_{i-1}\right)+2x_{i-1}\right)+x_{i-1},\frac{1}{2}\Delta \text{t}f\left(\frac{\Delta \text{t}}{2}+x_{i-1},\frac{1}{2}\Delta \text{t}x_{i-1}+x_{i-1},\frac{1}{2}\Delta \text{t}f\left(x_{i-1},x_{i-1},x_{i-1}\right)+x_{i-1}\right)+x_{i-1}\right)+f\left(x_{i-1},x_{i-1},x_{i-1}\right)\right)+\Delta \text{t}x_{i-1}, \end{array}$$

(57) $$\begin{array}{c} x_{i}=x_{i-1}+\frac{\Delta \text{t}}{6}\left(2\left(f\left(\frac{\Delta t}{2}+x_{i-1},\frac{1}{2}\Delta tx_{i-1}+x_{i-1},\frac{1}{2}\Delta tf\left(x_{i-1},x_{i-1},x_{i-1}\right)+x_{i-1}\right)+f\left(\frac{\Delta t}{2}+x_{i-1},\frac{1}{4}\Delta t\left(\Delta tf\left(x_{i-1},x_{i-1},x_{i-1}\right)+x_{i-1}\right)+2x_{i-1}\right)+x_{i-1},\frac{1}{2}\Delta tf\left(\frac{\Delta t}{2}+x_{i-1},\frac{1}{2}\Delta tx_{i-1}+x_{i-1},\frac{1}{2}\Delta tf\left(x_{i-1},x_{i-1},x_{i-1}\right)+x_{i-1}\right)+x_{i-1}\right)\right)+f\left(\Delta t+x_{i-1},\Delta t\left(\frac{1}{2}\Delta tf\left(\frac{\Delta t}{2}+x_{i-1},\frac{1}{2}\Delta tf\left(x_{i-1},x_{i-1},x_{i-1}\right)+x_{i-1}\right)+x_{i-1}\right)+x_{i-1},\Delta tf\left.\left.\left(\frac{\Delta t}{2}+x_{i-1},\frac{1}{2}\Delta tx_{i-1}+x_{i-1},\frac{1}{2}\Delta tf\left(x_{i-1},x_{i-1},x_{i-1}\right)+x_{i-1}\right)+x_{i-1}\right)+x_{i-1}\right)+f\left(x_{i-1},x_{i-1},x_{i-1}\right)\right). \end{array}$$

The above formulas are used to solve any i.v.p. having the form:

(58) $$\begin{array}{c} \ddot{x}=f\left(t,x,\dot{x}\right),x\left(0\right)=x_{0}\text{ and }x\prime \left(0\right)={\dot{x}}_{0}. \end{array}$$

The initial values for solving the recurrences (56)–(57) are *x*_0_ and ${\dot{x}}_{0}$.

## 8. Analysis and Discussion

Let us examine the accuracy of the obtained solutions. Let *ε*=0.1, *α*=*β*=1, *x*_0_=1, ${\dot{x}}_{0}=0$, and 0 ≤ *t* ≤ 35. The problem to be solved reads

(59) $$\begin{array}{c} \ddot{x}+0.2\varepsilon +x+x^{3}=0,\;x\left(0\right)=1\text{ and }x\prime \left(0\right)=0. \end{array}$$

The trigonometric solution is

(60) $$\begin{array}{c} x_{\text{trigo}}\left(t\right)=1.00251\text{exp}\left(-0.0938627t\right)\text{cos}\left(\begin{array}{c} 5.34045\left(\sqrt{3.96015+3.015082.71828^{-0.187725t}}+0.995006\;\log\left(0.502509\sqrt{3.96015+3.015082.71828^{-0.187725t}}-1.\right)-0.995006\text{log}\left(0.502509\sqrt{3.96015+3.015082.71828^{-0.187725t}}+1.\right)-0.68891+0.070781\right) \end{array}\right). \end{array}$$

The Jacobi solution is

(61) $$\begin{array}{c} x_{\text{Jacobi}}\left(t\right)=1.00227\text{ exp}\left(-0.0932826t\right)\text{cn}\left(\omega \left(t\right)\;|\;\frac{1}{2+1.97113\;\exp\left(0.186565\right)}\right), \end{array}$$

where

(62) $$\begin{array}{c} \omega \left(t\right)=-10.5079\sqrt{1.00455e^{-0.186565t}+0.990045}+10.4554{\text{tanh}}^{-1}\left(\frac{0.99501e^{0.0932826t}}{\sqrt{0.990045e^{0.186565t}+1.00455}}\right)+5.61143. \end{array}$$

**Figure 1:**
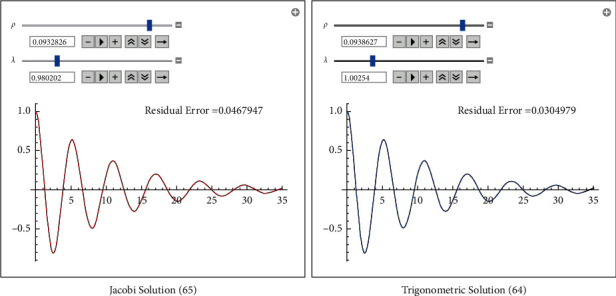
Approximate analytical solutions. Comparison between the numerical solution and the analytical approximate solutions-elliptic and trigonometric.

See Figure 1 for comparison with numerical solution.

## Figures and Tables

**Table 1:** Approximations for the elliptic integral $K\left(m\right)=\int_{0}^{\pi /2}\left(\text{d}\theta /\sqrt{1-m{\text{sin}}^{2}\theta }\right)$.

*K*(*m*)	Error on − 1 ≤ *m* ≤ 0.25
(30*m*/59)+(19/11)	0.169357
(11*m*^2^/26)+(23*m*/36)+(11/7)	0.0718427
(7*m*^3^/15)+(11*m*^2^/19)+(15*m*/43)+(35/23)	0.0503373
(19*m*^4^/32)+(19*m*^3^/28)+(2*m*^2^/29)+(5*m*/22)+(11/7)	0.0402712
(23*m*^5^/28)+(9*m*^4^/10) − (7*m*^3^/31) − (5*m*^2^/27)+(17*m*/41)+(51/32)	0.0232432
((*π*/2) − (5*πm*/32))/(1 − (9*m*/16))	0.000714295
(−(19*πm*^2^/1152) − (2*πm*/9)+(*π*/2))/(1 − (25*m*/36))	0.0000730467
((409*πm*^2^/9728) − (249*πm*/608)+(*π*/2)/(1025*m*^2^/4864) − (325*m*/304)+1)	3.85 × 10^−6^

**Table 2:** Approximate trigonometric solution to the i.v.p. (1) may be obtained in many ways.

1/*K*(*m*)	Error on − 1 ≤ *m* ≤ 0.25
(20/33) − (5*m*/28)	0.0322711
−(*m*^2^/13) − (8*m*/41)+(7/11)	0.0104322
−(2*m*^3^/31) − (2*m*^2^/21) − (2*m*/13)+(9/14)	0.0063857
−(3*m*^4^/44) − (3*m*^3^/35) − (*m*^2^/29) − (10*m*/71)+(7/11)	0.00418393
−(*m*^5^/12) − (2*m*^4^/21)+(*m*^3^/124) − (*m*^2^/95) − (5*m*/31)+(19/30)	0.00331487
((2/*π*) − (9*m*/8*π*))/(1 − (5*m*/16))	0.000251464
((19*m*^2^/160*π*) − (8*m*/5*π*)+(2/*π*))/(1 − (11*m*/20))	0.0000193383
((1025*m*^2^/2432*π*) − (325*m*/152*π*)+(2/*π*))/((409*m*^2^/4864) − (249*m*/304)+1)	1.36 × 10^−6^

## Data Availability

No data were used to support this paper.
